# Bioactive Compounds of Edible Fruits with Their Anti-Aging Properties: A Comprehensive Review to Prolong Human Life

**DOI:** 10.3390/antiox9111123

**Published:** 2020-11-13

**Authors:** Rajni Dhalaria, Rachna Verma, Dinesh Kumar, Sunil Puri, Ashwani Tapwal, Vinod Kumar, Eugenie Nepovimova, Kamil Kuca

**Affiliations:** 1 School of Biological and Environmental Sciences, Shoolini University of Biotechnology and Management Sciences, Solan (Himachal Pradesh) 173229, India; rajnidhalaria86@gmail.com (R.D.); spuri56@yahoo.com (S.P.); 2 School of Bioengineering and Food Technology, Shoolini University of Biotechnology and Management Sciences, Solan (Himachal Pradesh) 173229, India; chatantadk@yahoo.com; 3 Himalayan Forest Research Institute, Shimla H.P. 171009, India; ashwanitapwal@gmail.com; 4 School of Water, Energy and Environment, Cranfield University, Cranfield MK430AL, UK; Vinod.Kumar@cranfield.ac.uk; 5 Department of Chemistry, Faculty of Science, University of Hradec Kralove, Hradec Kralove 50003, Czech Republic; eugenie.nepovimova@uhk.cz

**Keywords:** bioactive compounds, anti-aging, edible fruits, life extension, antioxidants, free radicals, health benefits

## Abstract

Aging is a complicated biological process in which functional and structural alterations in a living organism take place over time. Reactive oxygen species is one of the main factors responsible for aging and is associated with several chronic pathologies. The relationship between aging and diet is quite interesting and has attained worldwide attention. Healthy food, in addition to dietary antioxidants, are required to delay the process of aging and improve the quality of life. Many healthy foods such as fruits are a good source of dietary nutrients and natural bioactive compounds which have antioxidant properties and are involved in preventing aging and other age-related disorders. Health benefits linked with healthy consumption of fruit have drawn increased interest. A significant number of studies have documented the advantages of fruit intake, as it suppresses free-radical development that further reduces the oxidative stress created in the body and protects against several types of diseases such as cancer, type 2 diabetes, inflammatory disorders, and other cardiovascular diseases that ultimately prevent aging. In addition, fruits have numerous other properties like anti-inflammatory, anti-cancerous, anti-diabetic, neuroprotective, and have health-promoting effects. Mechanisms of various bioactive compounds that aids in preventing various diseases and increases longevity are also described. This manuscript provides a summary of various bioactive components present in fruits along with their health-promoting and antiaging properties.

## 1. Introduction

Aging is a slow process of physiological deterioration that each living organism experiences with time. In fact, aging is a primary risk factor connected with considerably raised incidences of several degenerative diseases in particular type 2 diabetes, cancer, Alzheimer’s disease, cardiovascular disease (CVD), and these chronic diseases account for deaths among peoples [1]. Aging at the biological level is distinguished by the accumulation of cell and molecular damage resulting in functional and structural changes in tissues and cells, such as impaired intercellular contact, senescence, loss of mitochondrial homeostasis, and reduced regenerative ability [2]. Approximately 150,000 people across the world die every day of aging and around two-thirds die of age-related diseases [3]. Among several agents that are believed to play a significant part in the age-linked decline of functions, are free radicals that include reactive nitrogen species (RNS) and reactive oxygen species (ROS) and play a vital role [4,5].

Free radicals and reactive species are natural byproducts that are generated in organisms through both physiological and environmental processes [6]. Free radicals are normally generated as a result of ATP (adenosine triphosphate) production in the mitochondria when cells use oxygen (an essential element for life) to produce energy. Thus, an imbalance between overproduction or accretion of free radicals in the body and the potential of a biological system to detoxify the reactive substances results in oxidative stress, which is the leading factor in the development of several degenerative and age-linked chronic disorders [7,8]. A plethora of evidence proposed that the oxidative stress produced in mitochondria as a byproduct of cellular respiration is the main reason for aging [9]. Delay or inhibition in the pathogenesis of such diseases by plants is also an attractive strategy to encourage healthy aging [3]. So, a proper nutritional diet is recognized in combating these diseases as it has a substantial influence on aging and health without any side effects [10]. Furthermore, the optimistic connection between aging and diet has escalated the consumer interest in gaining more knowledge of a functional diet, rich in antioxidants, such as vegetables, fruits, and their related products [11,12,13,14]. Antioxidants are the natural substances present in fruits and vegetables that protect the cell from free radical damage by neutralizing and scavenging them. Among these, fruits are of great importance and have attracted worldwide attention from researchers due to their nutritional value, delicious taste, vitamins, minerals, and fiber content. Several research findings have also reported that healthy fruit intake is related to a lower prevalence of chronic diseases [15,16,17,18,19].

Various fruits and their derivatives are well-known to hold an elevated level of naturally occurring polyphenolic compounds [18,20,21,22]. Polyphenols are the plant secondary metabolites with antioxidant properties that function as free radical inhibitors and play a vital part in reducing oxidative stress that ultimately prevents aging and their associated diseases [23,24]. Moreover, several bioactive compounds such as catechins, anthocyanins, and isoflavones have potent antioxidant activity against ROS. Frequently consumed fruits, particularly apples, grapes, berries, oranges, and cherries contain different polyphenolic compounds that have a beneficial impact on human health [25,26]. The presence of high bioactive content in these fruits aid in delaying the processes of aging and alleviates the risk of various age-linked chronic disorders like CVD and cancer. Polyphenols, however, contain a broad diversity of compounds and are categorized into various groups like stilbenes, tannins, flavonoids (flavanones, flavanols, flavones, flavonols, isoflavones, proanthocyanidins, anthocyanins), phenolic acids, and lignans [27,28,29]. It has been documented that an extensive variety of antioxidant activities and phytochemical levels occur inside and across fruit genera [22,30].

Pterostilbene, resveratrol, and quercetin are the naturally occurring phytochemicals or the polyphenolic antioxidants present in a variety of fruits such as cranberries, bilberries, and blueberries (*Vaccinium* sp.) [31]. Recent findings have shown that these have positive effects such as anti-aging and a tendency to prolong lifespan by controlling the signs of aging, inflammation, cell senescence, oxidative damage, and telomeric attrition [32,33]. The main purpose of this manuscript is to develop an outline about various nutraceuticals and bioactive compounds present in fruits, and their anti-aging, and other health-promoting properties that increase life expectancy in humans.

## 2. Free Radicals and Aging

Aging refers to the universal, progressive, and deleterious changes in organisms that occur with time and which intensifies the probability of several diseases and sometimes leads to death [34]. Interestingly, chronic diseases and aging both are highly linked with DNA mutations, low-grade inflammation, and increased metabolic and oxidative stress, including escalated levels of its damage [3]. The human body is in a continuous fight to stay itself away from aging. One of the well-studied and most prominent theories about aging is the free radical theory of aging [5]. 

Free radicals are unstable, highly reactive, and self-existent molecules, comprising one or more unpaired electron in a nuclear orbit. They can either accept an electron or donate an electron to other molecules and thus serve as reductants or oxidants [35]. Free radicals are generally present in the body as a natural byproduct of chemical processes like metabolism that can upsurge the chances of various diseases and quicken the process of aging. ROS such as superoxide radical (O_2_^•^), peroxyl radical (ROO^•^), alkoxyl radical (RO^•^), hydroxyl radical (^•^OH), and RNS such as nitric oxide (NO) and nitrogen dioxide (NO_2_) are among the most common free radicals originating from both exogenous and endogenous sources [6]. Exogenous sources of ROS/RNS include environmental pollutants, radiations, industrial chemicals, drugs, xenobiotics, and smoke [35,36,37]. The endogenous sources include phagocytosis, inflammatory responses, and cellular metabolic processes such as mitochondrial electron transport [38,39].

Overproduction of ROS in the human body damages diverse biomolecules via redox reactions and leads to cellular damage, mutation, cell death, and aging [40,41,42,43]. ROS are also implicated in several chronic illnesses and other age-related disorders. Generally, two groups of antioxidants, viz. enzymatic and non-enzymatic antioxidants, regulate free radical reactions. The human body uses enzymatic antioxidant defense mechanisms to maintain the balance between free radicals and antioxidants by eliminating surplus ROS. The antioxidant enzymes minimize the level of H_2_O_2_ as it is essential in the preclusion of lipid peroxidation and retaining the structure and function of cell membranes. Various enzymatic antioxidant enzymes that are involved in free radical scavenging activity are superoxide dismutase (SOD), catalase (CAT), and glutathione peroxidase (GSHPx) as shown in the following reactions.

$$2O_{2}^{{•}_{-}}+2H^{+}\overset{\left(SOD\right)}{\to }H_{2}O_{2}+O_{2}$$

$$2H_{2}O_{2}\overset{\left(Catalase\right)}{\to }2H_{2}O+O_{2}$$

$$H_{2}O_{2}+2GSH\overset{\left(GSHPx\right)}{\to }2H_{2}O+GSSG$$

SOD are found in mitochondria and cytosol of the cell, catalytically convert the superoxide radical (O_2_^•−^) into hydrogen peroxide (H_2_O_2_) and oxygen (O_2_) in the presence of metal ion cofactors like zinc (Zn) and copper (Cu) [44]. The CAT enzyme located in peroxisome uses iron as a cofactor and catalyzes the reduction or degradation of hydrogen peroxide (H_2_O_2_) to form water (H_2_O) and molecular oxygen (O_2_), thus completing the detoxification process started by SOD [45]. GSHPx is an intracellular enzyme present mainly in mitochondria and cytosol, that breaks hydrogen peroxide (H_2_O_2_) into two water molecules (H_2_O) and oxidizes GSH (glutathione). The activity of GSHPx generally depends on the selenium [46].

Similarly, RNS such as nitric oxide (NO^•^) is produced in the human body from amino acid L-arginine in the presence of enzyme nitric oxide synthase (NOS) as shown in the equation:

$$L-Arginine+O_{2}+NADPH\overset{\left(NOS\right)}{\to }NO^{•}+Citrulline$$

Generation of free radicals occurs with the absorption of oxygen, the activation of NADPH oxidase, and the production of superoxide anion radicals is shown in the equation:

$$2O_{2}+NADPH\overset{\left(oxidase\right)}{\to }2O_{2}^{{•}_{-}}+NADP^{+}+H^{+}$$

The inducible nitrogen oxide synthase (iNOS) is involved in the synthesis of NO^•^ and reacts with oxygen radicals (O_2_)^•−^. The NO^•^ and O_2_^•−^ react together (radical-radical coupling) to yield peroxynitrite (ONOO^−^), which is a powerful oxidant that can attack a large range of biological targets [47].

$$NO^{•}+O_{2}^{•}\to ONOO^{-}$$

**Figure 1:**
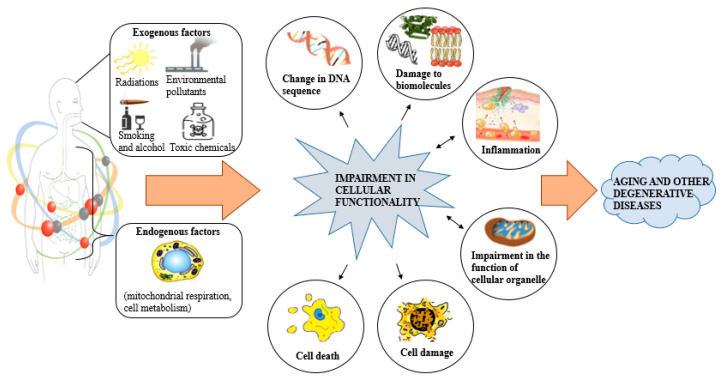
Mechanism of free radical formation and its impact on the cell. Modified from Khanthapok et al. [53] with permission.

Insufficient antioxidant defense systems and a great amount of ROS/RNS cause the accretion of free radicals in the cells, which causes oxidative damage [48]. Cellular oxidative stress causes protein dysfunction, loss of structural integrity, deleterious damage to the cell membrane, DNA, mitochondrial DNA contributes considerably to chronic age-linked diseases mainly CVD, type 2 diabetes, cancer, hypertension, and atherosclerosis as shown in Figure 1 [39,49,50,51,52].

## 3. Nutraceuticals and Bioactive Compounds in Fruits: Source of Antioxidants

Plants synthesize various phenolic compounds that are present in different parts of the plant, but particularly in fruits, leaves, and seeds, where they are mainly used to protect against pathogens and UV radiations [54,55]. Many foods (plant-based) in a healthy diet like fruits and vegetables contain most of the naturally occurring polyphenols [24]. Fruits are not merely a source of non-nutritive compounds containing phenolics but a great source of a large variety of nutritional compounds containing minerals (iron, copper, zinc, manganese, and selenium), vitamins (C, A, E), and dietary fibers [15,17,56,57]. These minerals and vitamins serve as antioxidants that help in reducing several chronic and age-related diseases, mainly diabetes, cancer, coronary heart disease, CVD, and provide beneficial health benefits and encourage healthy aging [55]. Antioxidants also help in reducing inflammation [58]. As dietary compounds present in fruits activate the nuclear erythroid-2 like factor-2 (Nrf2), a key regulator of antioxidants that inhibits the activation of NF-κB (nuclear factor kappa B) pathway which is involved in developing inflammation. Nrf2 increases antioxidant defenses, which efficiently neutralizes ROS by regulating toll-like receptor 4-mediated NF-κB activation [59,60].

### 3.1. Nutraceuticals

Nutraceuticals are natural foods including vitamins, minerals, and have physiological benefits that protect various chronic pathologies. Nutraceuticals help in delaying the process of aging, improve health, and support the structure and function of the body or increase life expectancy.

#### 3.1.1. Vitamins

Vitamin is a major micronutrient that the body requires for the proper functioning of its metabolism. Humans cannot naturally synthesize these nutrients in their bodies and try to meet their requirements through food sources that are rich in vitamins [11]. Various fruits such as oranges, berries, grapefruit, cherries, apples, etc. contain significant quantities of vitamins C, E, and A. These vitamins aid in boosting the immune system and decrease inflammation [22]. They also have powerful reducing properties which make them a better antioxidant and help in mitigating the effects of oxidative stress and contribute towards aging and associated diseases. Vitamin C (ascorbic acid), is water-soluble that acts as the first defense against free radicals and present in relatively high content in fruits like strawberries, oranges, and black currants (58.8, 53.2, and 41 mg per 100 g of fruit respectively) [61,62].

**Figure 2:**
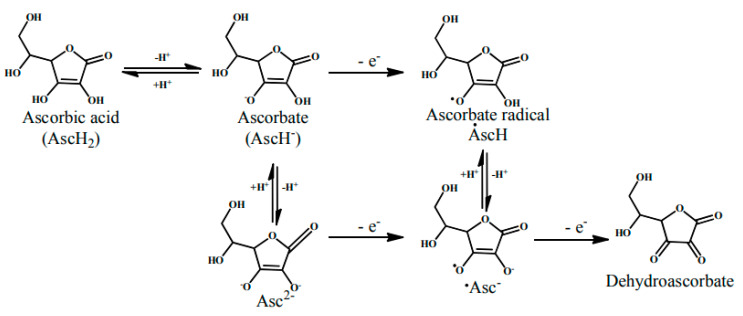
Mechanism of free radical scavenging activity of vitamin C [65].

Vitamin C is a potent antioxidant and radical scavenger that avert free radicals from damaging DNA, tissues, cell membranes [63,64] and regenerates vitamin E, a lipid-soluble vitamin in lipoproteins and membranes. Vitamin C (ascorbic acid) changes to the ascorbate radical by giving an electron to the lipid radical to stop the lipid peroxidation chain reactions described in Figure 2.

**Figure 3:**
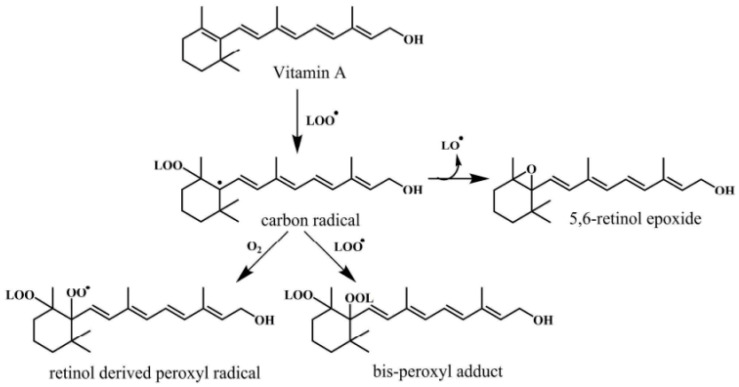
Mechanism of free radical scavenging activity of vitamin A [65].

The pairs of ascorbate radicals then react and form a molecule of dehydroascorbate and ascorbate. The dehydroascorbate does not possess any antioxidant potential so it is transformed back by the addition of two electrons into the ascorbate [63,65]. During lipid peroxidation, vitamin E act as a chain breaker in several lipid particles such as low-density lipoprotein (LDL) and in cell membranes. It functions to intercept lipid peroxyl radicals and to terminate the lipid peroxidation chain reactions [65]. The combination of ascorbic acid with α-tocopherol (Vitamin E) is mainly efficient in preventing oxidation [66]. Vitamin A is also a lipid-soluble vitamin that acts as an antioxidant and helps in scavenging free radicals to prevent a variety of chronic pathologies are described in Figure 3. Monaghan and Schmitt [67] first identified the antioxidant potential of vitamin A and stated that this vitamin can protect lipids from rancidity. Vitamin A also has a major antioxidant impact in protecting human LDL against copper-stimulated oxidation [65,68].

#### 3.1.2. Minerals

Minerals are those elements present on earth and in food that are required as essential nutrients for organisms to grow and perform various functions necessary for life. Fruits such as apples, berries, cherries, and grapes are abundant in both micro and macronutrients containing minerals. The key minerals present in these fruits are potassium, magnesium, calcium, phosphorus, iron, sodium, copper, zinc, selenium, and manganese. Berries accumulate a great deal of phosphorus, calcium, sodium, and iron minerals from the environment and hold supremacy over all other fruits [22]. Several microelements such as iron, selenium, zinc, copper, and manganese act as cofactors for various antioxidant enzymes and participate in redox metabolism which further helps in slowing the process of aging as they decrease ROS in cells, thus increasing the life expectancy of organisms [11,46]. Mineral nutrients are clinically acknowledged as necessary elements for consumer health since they provide strength to muscles and play a crucial part in the teeth and bones development. These major mineral elements are implicated in multiple essential biochemical and physiological processes that occur in humans. The mineral content of several fruits is shown in Table 1.

### 3.2. Bioactive Compounds

Bioactive compounds are important complexes, found in foods and are efficient in regulating different metabolic activities and results in better health [69,70]. Furthermore, several fruits constitute a broad diversity and large content of bioactive compounds particularly tannins, stilbenes, flavonoids, and phenolic acids [14,56,71]. Polyphenols play a vital part in fruits and are used as antioxidants and colorants [72]. Intake of dietary antioxidants helps to uphold an adequate antioxidant status in the human body. A substantial amount of research on polyphenols is emphasized on their antioxidant properties after they are believed to have optimistic effects on age-related chronic pathologies. Various studies have also reported that polyphenols rich diet can prevent oxidative damage leading to aging [73]. Fruits like berries, cherries, apples, and grapes constitute approximately 200–300 mg polyphenols per 100 g of fresh weight [24,74]. The products derived from these fruits, constitute a great proportion of polyphenols. Several polyphenols including catechin, epicatechin, rutin, proanthocyanidin B2, phloretin glycosides, quercetin glycosides, and chlorogenic acid are mostly found in apples which have a strong antioxidant property [11]. Different polyphenols with antioxidant properties that are found in different fruits are shown in Table 2.

These molecules can act as an antioxidant (in-vivo) in various ways: (i) by scavenging reactive species due to an elevated reactivity (measured as a rate constant) that allows it to scavenge oxidants before they can affect other biological targets such as nucleic acids and proteins; (ii) by inducing endogenous antioxidant responses through Nrf2-dependent gene expression to modulate the pathophysiological and physiological outcomes of oxidant exposure [96]; (iii) by inhibiting the production of ROS/RNS either by inhibiting the expression or activities of enzymes such as NADPH oxidases or xanthine oxidase, inhibiting inflammation or by decreasing mitochondrial electron leaking [97]. A study published in 2008, which investigated the effect of dietary supplementation with red grape juice (source of vitamin E and polyphenols) on neutrophil NADPH oxidase activity and cardiovascular risk factors in thirty-two patients on hemodialysis. The findings suggested that both red grape juice and vitamin E reduced ex-vivo neutrophil NADPH oxidase activity and plasma concentrations of oxidized LDL. Red grape juice also causes a reduction in cardiovascular risk factors [98]. Thus, findings indicate that natural antioxidants are the possible inhibitors of NADPH oxidase.

#### 3.2.1. Phenolic Acids

Phenolic acid is abundantly present in fruits and is separated into two main classes: hydroxybenzoic acid and hydroxycinnamic acid. Most of the berries, particularly blackberries, raspberries, blueberries, cranberries, apples, oranges, and cherries are rich in both hydroxybenzoic and hydroxycinnamic acid [99,100]. The most prevalent hydroxybenzoic acids are vanillic, syringic, gallic, protocatechuic, and p-hydroxybenzoic acids, while the corresponding hydroxycinnamic acids are sinapic, p-coumaric, ferulic, and caffeic acids. These derivatives vary in methoxylations and hydroxylations patterns of their aromatic rings. Phenolic acids are mostly present in bound forms and serve as a potent antioxidant because of the reactivity of phenol moiety; a hydroxyl substituent on the aromatic ring [101]. A few derivatives of hydroxybenzoic acids are presently used as additives to decrease nutrient oxidation and to improve the nutritional value of fruits. A wide array of phenolic compounds have the potential to scavenge ROS including hydroxyl radicals and superoxide radicals, which decreases lipid peroxyl radicals and prevents lipid peroxidation. Phenolic acids act as a powerful anti-radical agent because of their redox properties, which makes them efficient hydrogen donors and metal chelators [101]. The phenolic content of several fruits is listed in Table 3.

Research on the structural activity of phenolic acids and their derivatives revealed that the derivatives of hydroxycinnamic acid had greater antioxidant ability in comparison to that of benzoic acid counterparts [104]. This potential was because of the existence of a propanoic side chain in cinnamic derivatives; the conjugated double bond in their side chains has an intense effect on the phenoxyl radical by resonance, thereby improving the antioxidant power. Gallic acid is also a powerful antioxidant, found abundantly in strawberries, raspberries, red grapes, grapefruit, cranberries, blackberries, also in juices made from these fruits [75]. Antioxidant activity of gallic acid is triple times more than that of vitamin E or C, representing that three hydroxyl groups of gallic acid can independently act as electron acceptors [77]. Derivatives of gallic acid, thus, also act as a strong antioxidant with a free hydroxyl group that is responsible for radical scavenging and apoptosis of cancer cells. The most intriguing benefits of gallic acid have been evaluated on the skin [105]. In the case of prostate cancer cells, gallic acid prevents multiplication and the death of cells. Phenolic acids have been reported to possess various useful therapeutic activities like anti-inflammatory, anti-viral, anti-bacterial, anti-allergic, anti-cancer, anti-mutagenic, and anti-melanogenic activities [77,106]. Ruifeng et al. [107] conducted an experiment to evaluate whether chlorogenic acid could ameliorate the inflammation response in lipopolysaccharide-induced mice mastitis. The findings revealed that chlorogenic acid significantly decreased the production of tumor necrosis factor-alpha (TNF-α), interleukins (IL-1β, IL-6) against lipopolysaccharide-induced mastitis. The western blot analysis indicated that chlorogenic acid could suppress the expression of toll-like receptor (TLR4), the phosphorylation of nuclear factor kappa B (NF-κB), and the inhibition of NF-κB (IκB) induced by lipopolysaccharide, and thus highlights the anti-inflammatory response.

#### 3.2.2. Flavonoids

Flavonoids are diverse and the most studied group of polyphenols found abundantly in several fruits like blackberries, blueberries, raspberries, blackcurrants, strawberries, grapes, cranberries, apples, cherries, etc. Currently, more than 8000 flavonoids are recognized, some of which are responsible for the fascinating colors of the leaves, flowers, and fruits [108]. Phenolic compounds obtained from natural sources are considered much safer in terms of having no side effects than synthesized chemicals [109,110]. Synthetic antioxidants are involved in triggering diseases in humans beyond certain concentrations [111]. In fruits, flavonoids are found in glycosides or acyl glycosides form, while methylated, acylated, and sulfate molecules are rare and present in low concentrations. The flavonoid content in various fruits is listed in Table 3. The common basic structure of flavonoids comprises two aromatic rings that are joined to each other by three carbon atoms and forms a closed oxygenated heterocyclic pyran ring [112]. Flavonoids are classified into six subclasses based on differences in the type of heterocycle present in this group: flavanols (blueberries and apples), flavonols (grapes), flavanones (citrus fruits), flavones, isoflavones, and anthocyanins (grapes and berries) [113,114]. Flavonols, flavanols, and anthocyanins are ubiquitous and possess strong antioxidant properties, which mainly depends on the position and number of hydroxyl groups present within their structure. Flavonoids also exert a wide variety of biological actions namely antibacterial, antioxidant, anti-inflammatory, anti-hyperlipidemic, and hepatoprotective activities [115].

Flavonoids in citrus can suppress phosphodiesterase and kinases activation implicated in the initiation phase of inflammation. These enzymes can affect protein kinases and proinflammatory TNF-α expression. Some flavonoids can suppress the induction of endothelial cell adhesion molecules that are activated by cytokines. Inflammatory responses are also suppressed by impeding the monocytes, leukocytes, and neutrophils adhesion from injured regions, thus highlighting the anti-inflammatory impact [116,117]. Flavonoids also reduce the risk of coronary heart disease by averting low-density lipoproteins (LDLs) from oxidizing, reducing the ability of the platelets in the blood to clot, and by improving coronary vasodilatation [118]. Flavonoids can act in various developmental stages of malignant tumors by inactivating carcinogens, protecting DNA against oxidative damage, suppressing mutagenic genes, and enzymes expression accountable for triggering pro-carcinogenic substances, and triggering the systems accountable for xenobiotic detoxification [119]. Most of the studies have shown a structural-functional relationship, indicating that anti-proliferative, enzyme-inhibition, and antioxidant activities of flavonoids are reliant upon specific structural motifs [120,121,122].

##### Anthocyanins

Anthocyanins are the pigments that usually occur in nature, inhabiting a unique position in the group of polyphenols [123]. They are broadly distributed in a large number of fruits and vegetables and a great concentration of anthocyanins is observed in cranberries, blackcurrants, strawberries, bilberries, raspberries, blackberries, blueberries, and chokeberries [124,125]. They also constitute an immense group of colored water-soluble pigments that give the fruit purple, blue, and red colors [55]. However, anthocyanins are not solely responsible for the fruit color but are often used as a natural pigment in the food industry. Over 600 anthocyanins have been recognized in nature to date, but only six anthocyanins are widely found in fruits: malvidin, pelargonidin, peonidin, delphinidin, cyanidin, and petunidin [126,127]. The overall anthocyanin content in several fruits is mentioned in Table 3. Anthocyanins are also known as effective natural antioxidants [128]. The chemical structure of anthocyanin determines its efficacy as an antioxidant agent. The antioxidant activity of anthocyanin is allied with free hydroxyl numbers around the pyrone ring. Larger the hydroxyl number larger the antioxidant activity [129]. The anthocyanins have indicated characteristics that suppress free radical formation, reduces the threat of various age-linked diseases like cancers, CVD, improves aging and memory [130]. In the case of fruits, the anthocyanins are found mostly in the outer layer of the pericarp. Cyanidin-3-glucoside is the main anthocyanin that is present in maximum fruits. Anthocyanins include aglycones and their glycosides—anthocyanidins and anthocyanins, and also form different complexes [131].

Anthocyanins vary in terms of the hydroxyl groups number in a molecule, their methylation degree; place, form, and number of sugar molecules attached, number, and form of aromatic and aliphatic acids attached to sugars. In berries, anthocyanins are present in different glycosides forms i.e., mono-, di- or tri-, where residues of glycosides are commonly substituted at C3 or rarely, at C5 or C7 position [56]. The most predominant sugars are sophorose, sambubiose, rutinose, arabinose, rhamnose, galactose, and glucose [132]. The glycoside residues of anthocyanin are frequently acylated by ferulic, p-coumaric, caffeic acid, and by acetic or malonic acid, p-hydroxybenzoic acid [131,133].

Anthocyanins exert several biological properties like anti-tumor, anti-inflammatory, antioxidant, anti-diabetic, anticancer, and neuroprotective [134]. A researcher [135] experimented to assess the chemopreventive effect of anthocyanin-rich black currant skin extract with concentrations 100 and 500 mg/kg against diethyl nitrosamine-initiated hepatocarcinogenesis in rats for 18 weeks. The findings showed a decrease in iNOS expression, 3-nitrotyrosine, abnormal lipid peroxidation, and protein oxidation in a dose-dependent manner. Mechanistic studies have shown that black currant skin extract upregulated the gene expression of a number of carcinogens detoxifying and hepatic antioxidant enzymes, like uridine diphosphate-glucuronosyltransferase isozymes, glutathione S-transferase and NAD(P)H:quinone oxidoreductase in diethyl nitrosamine-initiated animals. This treatment significantly raised mRNA and protein expressions of nuclear factor E2-related factor 2 (Nrf2), providing evidence of a synchronized activation of the Nrf2-regulated antioxidant pathway, which results in the activation of multiple housekeeping genes. Moreover, the anti-inflammatory activity of anthocyanins can be attributed to their antioxidant property which results in the down-regulation of the redox-sensitive nuclear factor-κB signaling pathway [136].

##### Catechins

Catechin is a polyphenolic antioxidant present in several fruits such as blueberries, strawberries, gooseberries, cherries, black grapes, and apples. Catechin term refers to the flavonoid class and flavan-3-ols/flavanols sub-class. The most important dietary catechins are gallocatechin, epicatechin (EC), epicatechin 3-gallate (ECG), epigallocatechin 3-gallate (EGCG), and epigallocatechin (EGC). In strawberry, 2–50 mg/100 g total amount of catechins is present. Cherries contain 5–22 mg/100 g catechins whereas apples contain 10–43 mg/100 g catechins but both are abundant in EGCG [137]. Catechins are rich in external tissues of the fruit and are distinguished from the flavonoids containing ketones, in particular rutin and quercetin, which endorse the antioxidant defense system. Catechin present in cranberries is like the catechin present in green tea, that aids in protection against cancer [22]. The antioxidant action of catechin is effective against cancer, neurodegenerative and cardiovascular diseases [138]. A study conducted by researchers [139] reported that epigallocatechin gallate (EGCG), a major catechin strongly inhibits an enzyme called telomerase, which is necessary for unlocking the proliferative capacity of cancer cells by upholding the tips of their chromosomes. Thus, it may be another reason for the anticancer activity of catechins. The antioxidant property of catechin proved beneficial in reducing coronary heart diseases as it decreases cytotoxicity caused by amiodarone in fibroblast cells of the lungs [140]. Catechins also show anti-inflammatory activities in bowel disease in humans by effecting oxidative stress-related cell signaling pathways, such as transcription factor nuclear factor (erythroid-derived 2)-like 2 (Nrf2), mitogen-activated protein kinases (MAPKs), nuclear factor-kappa B (NF-κB), signal transducer and the activator of transcription 1/3 (STAT1/3) pathways [141]. Pan et al. [142] experimented to study the anti-inflammatory effects of catechins in an in vitro experiment using stimulated human nasal epithelial cells (HNEpCs) and in an ovalbumin-induced allergic rhinitis murine model. The results showed that catechin inhibited the expression of Thymic stromal lymphopoietin (a molecule that plays a main role in the development of allergy) in epithelial cells by influencing the NF-κB/TSLP pathway. Catechin effectively decreased the inflammation in allergic rhinitis. Catechins also reduce the degeneration of neurons by directly or indirectly decreasing oxidative stress, scavenging ROS, and improving antioxidant enzymes [140]. Thus, most of the benefits of catechins are achieved through the way of their antioxidant mechanism.

##### Quercetin

Quercetin is a natural polyphenolic compound, found abundantly in several fruits such as apples, grapes, blueberries, raspberries, cherries, and blackcurrant [82,143]. It is reported as one of the most powerful ROS scavengers in the flavonoid class and flavonol sub-class [144]. An experiment was proposed [145] to evaluate the free radical scavenging activity of flavonoids in H_2_O_2_ treated human myelogenous leukemia (K562) cells. The experimental findings revealed that quercetin and luteonin have shown the highest protective effects as compared to rutin, an apigenin towards H_2_O_2_ induced damage in leukemia cells. The antioxidant property of quercetin to scavenge free radicals is due to the presence of two antioxidant pharmacophores inside molecules that have the ideal structure for free radical scavengers [33,146]. Chondrogianni et al. [147] described in his studies that quercetin and its derivative quercetin caprylate (QU-CAP) can revitalize senescent human fibroblasts and extend their life expectancy by activating proteasome. It acts as an effective antioxidant that has significant pharmacological, biological, and medicinal properties. The antioxidant potential of quercetin is directly proportional to the number of free hydroxyl groups [148]. Moreover, it also exhibits potential anticancer and anti-inflammatory properties. Quercetin suppresses the production of inflammatory enzymes like lipoxygenase (LOX) and cyclooxygenase (COX) thus reducing the production of prostaglandins and leukotrienes, chemicals that promote inflammation [149]. The anti-inflammatory and antioxidant activities of quercetin and its derivatives contribute to the anti-aging effect since inflammation and chronic oxidative stress are considered to play a substantial part in activating the aging process [3].

#### 3.2.3. Tannins

Tannins are the essential constituents that play a major part in delineating the sensory characteristics of fruits and their products. They include both ellagic acid or esters of gallic acid called hydrolyzable tannins, and condensed tannins called proanthocyanidins [150]. They are responsible for variations in the fruit color and tart taste [151]. Tannins stabilize anthocyanins, in fruits rich in it, by binding them to form copolymers. Hydrolyzable tannins are rarely found and have been present in blackberries, blueberries, strawberries, and raspberries [82,95].

Ellagic acid contains approximately 51% of the total phenolic content and occurs in free as well as in complex form as glucosides and ellagitannins esterified as glucose. The presence of ellagic acid and its derivatives makes the berries (cranberries, raspberries, blackberries, strawberries) and grapes consumption appropriate for possible health benefits. In addition, the ellagic acid has been described to possess potent antiviral, anti-inflammatory, anti-proliferative, antioxidant properties and also provide defense against cancer of the esophagus, lungs, and colon [152,153]. Ellagic acid has been identified in several fruits in which the total concentration of ellagic acid was computed by evaluating the concentration after acid hydrolysis of ellagic acid extracts [22]. In the case of raspberries, free ellagic acid contains a small portion of total ellagic acid, and the main source ellagitannins is discharged by acid hydrolysis [95].

#### 3.2.4. Stilbenes

Stilbenes are low-molecular-weight compounds found in several fruits like berries and grapes. It comprises two phenyl moieties that are linked by a two-carbon methylene bridge. Generally, stilbenes are present in low concentrations in the human diet. One of the naturally occurring and most studied polyphenol stilbenes is resveratrol [154].

##### Resveratrol

Resveratrol (RES) is a phytoalexin that is formed by a broad diversity of plants as a response to fungal infections, injury, UV radiation, and stress. In 1940, the first resveratrol molecule was isolated from the *Veratrum grandiflorum* O. Loes roots (white hellebore) and later from berry-skins, grapevines, and *Vitis* spp. (grapes) leaves comprising their frequent and most examined source [31,155]. Research suggests that the resveratrol molecule is one of the main factors of the French Paradox that defines an epidemiological observation that connects fewer incidences of cardiovascular diseases and longer lifespan (despite a high-fat diet) in French people with the daily consumption of red wines [3,156,157]. As red wine comprises a substantial quantity of resveratrol, it has dragged great interest in the experimental community to explore its possible health benefits, whether the resveratrol might bestow these benefits.

Resveratrol is a polyphenolic antioxidant that belongs to the family stilbene and is synthesized by grapevine and various other plants as a response to pathogens attack from the precursor molecules of p-coumaroyl CoA and malonyl-coenzyme A (CoA), in the existence of enzyme stilbene synthase [158]. Resveratrol is found in various fruits like grapes and *Vaccinium* spp. (cranberry, bilberry, and blueberry). It has also been identified in plum fruit, increasing its natural wealth profile, particularly in dietary sources [31,159].

A plethora of evidence proposed that supplementation of dietary resveratrol exerts advantageous effects on aging and various other age-related chronic diseases in particular Alzheimer’s disease, cancer, and diabetes, etc. [160]. In addition, it was found to prolong lifespan by 70% in *Saccharomyces cerevisiae*, a typical model for aging studies. Over the past two decades, resveratrol has been the subject of extraordinary studies due to its anti-aging properties. It works both as a radical scavenger, as a chelating agent, and possesses anti-inflammatory activity [161].

## 4. An Insight into Pharmacological and Biological Benefits of Fruits

There is growing evidence from worldwide research that fruits are an imperative part of a balanced diet. Numerous phytochemicals from the fruits have antioxidant properties that safeguard against the detrimental effects of free radicals, which further lead to chronic pathologies linked with aging [162,163]. Fruits are also a good source of naturally occurring antioxidants which include vitamins, minerals, flavonoids, and phenolic acids. Presence of these antioxidants, aid in delaying or preventing oxidative damage of biomolecules by ROS which contain reactive free radicals including hydroxyl, peroxyl, alkoxyl, superoxide, and non-radicals such as hypochlorous, hydrogen peroxide, etc. The anthocyanins in fruits are also the most studied phenolic with a large variety of bioactivities including anti-aging, anticancer, anti-inflammatory, and antioxidant properties. Antioxidants scavenge these radicals by counteracting the formation of free radicals by attaching to metal ions or suppressing the initiation and chain-breaking propagation, by quenching superoxide and singlet oxygen, and by reducing hydrogen peroxide [164] and thus circumventing several age-linked diseases like type 2 diabetes, inflammation, cancer, and CVD. Various pharmacological and biological properties of several fruits are described in Table 4.

### 4.1. Anti-Aging Effects

Several vivid fruits and vegetables are extensively tested for their potential to neutralize free radicals that can destroy DNA and cell membranes via a mechanism called oxidative stress, which is responsible for most aging-related malfunctions and disorders [11]. While several fruits like blueberries, grapes, cranberries themselves are not a remedy for all of them, they contain a variety of substances such as vitamins, fibers, and antioxidants that have health benefits [14,22]. Antioxidants in fruits appear to have the most credible role in delaying or preventing aging, heart disease, cancer, and other age-related disorders. Clinical reports by nutritionists from laboratories across the world conclude that consuming raspberries is associated with health benefits [179]. Research conducted in 2018, investigated the effects of blueberry supplementation on lifespan and stress tolerance in *Caenorhabditis elegans.* The result showed that the mean lifespan of *Caenorhabditis elegans* was increased significantly on the dose-dependent basis by 22.2%, 36.5%, and 44.4%, respectively, when treated with blueberry extract at 50, 100, and 200 mg/mL for 4 days. In addition, blueberry treated *Caenorhabditis elegans* shows more resistance to stresses (heat, paraquat, and Ultraviolet-B radiation) than non-treated *Caenorhabditis elegans* [180]. Several experiments and trials with the various fruit extracts are displayed in Table 5.

### 4.2. Antioxidant Effects

Antioxidants are the substances that retard or prevent oxidation of a substrate while present in low concentrations. They have been of great interest to biochemists and healthcare professionals as they aid in protecting the body from the damage caused by ROS and RNS [219]. Various phytochemicals, such as phenolics and flavonoids, are the most active dietary antioxidants that help in safeguarding cells against oxidative stress induced by free radicals [220]. Nutritional antioxidants work by various mechanisms in different compartments, they are primarily free radical scavengers: as they minimize peroxide concentrations and overhaul oxidized membranes; directly neutralize free radicals; quench iron to reduce the development of ROS, through lipid metabolism in which short-chain free fatty acids and cholesteryl esters counteract ROS [221]. Recently, more focus has been paid to the health benefits of fruit consumption. As antioxidants present in fruits help in protecting the human body from the detrimental effects of free radicals associated with aging. Orena et al. [59] performed an experiment to assess the antioxidant response element (ARE) activation capacity of vegetable and fruit extracts in human IMR-32 cells. The findings showed that the fruit components activate the antioxidant response element. Fresh fruits constitute various natural antioxidants like phenolic, resveratrol, anthocyanins, vitamins (C, E) which show antioxidant properties and prevent oxidation of low-density lipoprotein (LDL) [131].

### 4.3. Anti-Inflammatory Effects

Inflammation is a physiological process of the body’s response to various kinds of injuries, but the deregulation of the inflammatory systems can cause severe damage to the cells and tissues of the host and contribute to disease development. Chronic inflammation is associated with multiple diseases, which include Alzheimer’s disease, CVD, metabolic syndrome, cancer, type 2 diabetes, and atherosclerosis [222,223]. Significant studies indicate that the intake of a fruit-rich diet is related to a reduced risk of inflammation-associated diseases. In-vitro and in-vivo research have highlighted the potential beneficial effects of fruits on chronic inflammatory diseases [26,224]. Cai et al. [225] in his research stated that supplementation of cranberry decreases the colonic levels of pro-inflammatory cytokines in mice treated with dextran sodium sulfate. Phytochemicals in raspberry effectively modulate gene expression, enzyme activity, and cellular pathways. In addition, to reduce the production of oxidized-LDL through their antioxidant activity, raspberry phytochemicals have shown anti-inflammatory and anti-atherosclerotic properties that protect against CVD [22]. The systematic consumption of polyphenol-rich foods, strawberry, or antioxidant has been shown to prevent inflammatory and fibrinolytic factors in foods [167,226].

### 4.4. Anticancer Effects

Cancer is a major health issue and almost 10 million people around the world die every year from cancer. A significant amount of epidemiological evidence indicates a strong connection between the dietary intake of fruit and the lower prevalence of several forms of cancers [227]. Several in-vitro and in-vivo studies have shown that polyphenolic extracts (triterpenoids, proanthocyanidin oligomers, and flavonols) from cranberries and their derivatives suppress the growth and multiplication of lung, prostate, colon, breast, and other tumors [228]. Many research findings have also revealed that the intake of a diet rich in phytochemicals including fresh vegetables and fruit leads to a decrease in the incidence of various types of human cancers [99]. Berries have demonstrated genoprotective, antioxidative, and anti-carcinogenic properties in different types of cancer cells and animal models. It has been evaluated that raspberry phytochemicals have the potential to suppress the in-vitro growth rate and proliferation of cancer cells by persuading S-phase arrest via the PTEN/AKT pathway [201]. Various other berries such as strawberry, blueberry, and cranberry have been shown to suppress cancer development by regulating numerous pathways, such as Wnt/β-catenin, ERK/MAPK, JAK/STAT, NF-κB, and PI3K/AKT/mTOR [167,229,230,231], thus changing cellular processes implicated in growth, multiplication, and metastasis. Berries inhibit the initiation of tumors by influencing the metabolism of carcinogens, leading to decreased rates of carcinogen-induced DNA damage. They prevent promotion events by decreasing the tissue inflammatory parameters and the progression rate of pre-malignant cells, encouraging apoptosis, and preventing angiogenesis [232]. Fruits polyphenols confer anticancer benefits through number of mechanisms, including the activation of metabolizing enzymes, gene expression modulation, and their influence on cell multiplication, cell signal transduction pathways, and cell death. Multiple studies have also indicated that the fruits have anti-cancer property and some of which are described in Table 5.

### 4.5. Neuroprotective Effects

Neurodegenerative diseases are widely known as significant health problems and aging is the primary risk factor for these diseases. Various epidemiological data have shown strong correlations between the intake of bioactive compounds, antioxidants, and decreased rates of neurodegenerative pathologies, such as aging [167,233]. As antioxidants obtained from natural sources help in combating various types of stresses one of which is migraine, which is considered a neurodegenerative disorder and helps in preventing the production of oxidative stress by suppressing the activation, spread, and oxidative chain reactions [234]. Clinical trials have also shown that the consumption of *Citrus* sp. is effective in reducing the risk of migraine attacks [235]. In 2006, Galli et al. [206] proposed an experiment on thirty male rats to study the effect of blueberries for 10 weeks. The findings showed that the short-term stimulation/exposure of blueberries results in enhanced HSP70-mediated defense against a variety of neurodegenerative activity in the brain. Numerous observations (in-vitro and in-vivo) indicate that plant phenols (flavonoids and phenolic acids) have a potential effect on neurological dysfunctions, and high consumption of antioxidants appears to be actively involved in enhancing neurological functions [236]. A research study observed correlation between cognitive decline and prolonged berry consumption, suggesting that a higher intake of berries is connected with a slower rate of cognitive decline in aged people [237]. Several other experiments with the various fruits extracts are displayed in Table 5.

### 4.6. Anti-Diabetic Effects

Diabetes mellitus is a rising problem for human wellness, with presently 463 million patients suffering from diabetes worldwide. This condition is caused by persistent high blood glucose levels (hyperglycemia), which are accountable for the dysfunction and failure of several organs. Various epidemiological studies have shown a link between increased fruit consumption, especially berries, and decreased diabetic incidences [238,239]. Research studies indicated that intake of strawberries, or their purified fraction of flavonoid, suppressed the transport and uptake of glucose and balanced their level in blood. As increased intake of fruit is significantly associated with a lesser incidence of diabetes in the prospective longitudinal cohort study. The roles of strawberry-flavored drink and strawberry freeze-dried beverage consumption were investigated in subjects with type 2 diabetes, evaluating the blood pressure and lipid profiles [167,240]. Significant reductions in total cholesterol (TC) levels, blood pressure levels, and TC to high-density lipoproteins (HDL) cholesterol ratios were found and compared to the initial values. The findings indicated that certain threat factors for CVD in subjects with diabetes were improved by the intake of strawberries over a short period of time [226]. Erlund et al. [241] also described that supplementation of only moderate amounts of berries resulted in positive improvements in platelet function, HDL cholesterol, and blood pressure. The findings show that the intake of berry plays a significant part in controlling CVD. In 2006, Ruel et al. [212] experimented on thirty abdominally obese men to observe the effect of consuming cranberry juice cocktails over a span of four weeks with an increase in daily doses (125, 250, and 500 mL). The results showed a rise in plasma HDL-cholesterol concentration, indicating a positive outcome in the reduction of the risk factor for CVD. Similarly, a study published in 2016 by Snyder et al. [194] which investigated the effect of apple peel extract and cherry extract on male mice for 10 weeks. The findings showed a large decrease in the amount of C-reactive protein in blood glucose. Various other research showing the anti-diabetic effect of fruit consumption are shown in Table 5.

## 5. Conclusions

As there is an increase in the prevalence of oxidative stress-mediated aging and other associated chronic pathologies, there is a need to improve the body’s defense mechanism with the least side effects. This need has been recognized by using potent antioxidants (naturally occurring compounds). In this regard, polyphenolic compounds in the fruit have demonstrated beneficial activity as antioxidants. Many natural antioxidants are highly effective in controlling the oxidative damage generated by free radicals and serve as a free radical scavenger. Various fruits like apples, oranges, berries, grapes, and cherries are a rich source of natural antioxidants like anthocyanins, phenolic, vitamins, minerals, and other anti-aging phytochemicals such as resveratrol, quercetin that are actively involved in postponing the process of aging and have health encouraging properties. Research on these fruits is of great importance to both the scientific and the public community. However, mechanisms that are linked with anti-aging in humans still need a detailed understanding of newly discovered phytochemicals.

## Figures and Tables

**Table 1:** Nutritional composition of various antioxidant-rich fruits [62].

Fruit	Macronutrients (in g)	Mineral Composition (mg/100 g)									
		P	K	Mg	Ca	Fe	Zn	Na	Cu	Mn	Se
*Citrus sinensis* 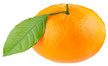	Carbohydrates = 11.75 Fats = 0.12 Proteins = 0.94 Dietary fibers = 2.4	14	181	10	40	0.1	0.07	0	0.045	ND	0.0005
*Fragaria x. ananassa* 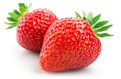	Carbohydrates = 7.68 Fats = 0.3 Proteins = 0.67 Dietary fibers = 2	24	153	13	16	0.41	0.14	1	0.048	ND	0.0004
*Malus pumila* 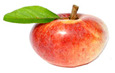	Carbohydrates = 13.81 Fats = 0.17 Proteins = 0.26 Dietary fibers = 2.4	11	107	5	6	0.12	0.04	1	0.027	ND	0
*Prunus avium* 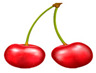	Carbohydrates = 16.01 Fats = 0.2 Proteins = 1.06 Dietary fibers = 2.1	21	222	11	13	0.36	0.07	0	0.06	ND	0
*Ribes nigrum* 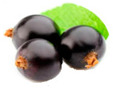	Carbohydrates = 13.8 Fats = 0.2 Proteins = 1.4 Dietary fibers = 4.3	44	275	13	33	1	0.23	1	0.107	ND	0.0006
*Rubus fruticosus* 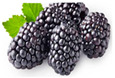	Carbohydrates = 9.61 Fats = 0.49 Proteins = 1.39 Dietary fibers = 5.3	22	162	20	29	0.62	0.53	1	0.165	ND	0.0004
*Rubus idaeus* 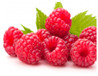	Carbohydrates = 11.94 Fats = 0.65 Proteins = 1.2 Dietary fibers = 6.5	29	151	22	25	0.69	0.42	1	0.09	0.67	0.0002
*Vaccinium corymbosum* * 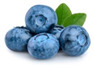 *	Carbohydrates = 14.49 Fats = 0.33 Proteins = 0.74 Dietary fibers = 2.4	12	77	6	6	0.28	0.16	1	0.057	ND	0.0001
*Vaccinium macrocarpon* 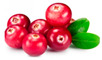	Carbohydrates = 11.97 Fats = 0.13 Proteins = 0.46 Dietary fibers = 3.6	11	80	6	8	0.23	0.09	2	0.056	ND	0.0001
*Vitis vinifera* 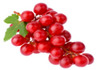	Carbohydrates = 18.1 Fats = 0.16 Proteins = 0.72 Dietary fibers = 0.9	20	191	7	10	0.36	0.07	2	0.127	0.071	0.0001

P = Phosphorus; K = Potassium; Mg = Magnesium; Ca = Calcium; Fe = Iron; Zn = Zinc; Na = Sodium; Cu = Copper; Mn = Manganese; Se = Selenium; ND = not defined.

**Table 2:** Dietary sources of polyphenols in fruits.

Category	Main Class	Sub-Class	Chemical Compound	Chemical Structure	Fruit Resources	References
Phenolic compound	Phenolic acids	Hydroxybenzoic acids	Gallic acid	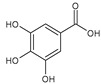	Strawberry, red grapes, raspberry, cranberry, blackberry	[75,76,77]
			Vanillic acid	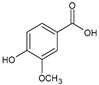	Strawberry	[78]
			Syringic acid	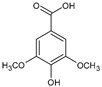	Strawberry	[78]
		Hydroxycinnamic acids	Chlorogenic acid	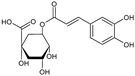	Apples, blackcurrant, blueberry, orange, cherry	[79,80,81,82]
			Caffeic acid	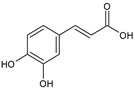	Strawberry, orange, cherry, blackberry	[83,84,85]
			Ferulic acid	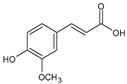	Orange	[84]
			p-coumaric	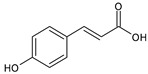	Strawberry, orange, blackcurrant, cranberry, cherry, blackberry	[81,82,84,86]
	Flavonoids	Flavonols	Quercetin	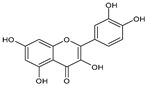	Apples, raspberry, blackcurrant, grapes, blueberry, blackberry	[80,82,87]
			Rutin	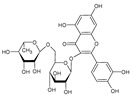	Apples, cherry, red grapes, oranges, blueberry, blackcurrant, raspberry	[82,83,87]
			Kaempferol	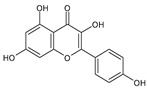	Strawberry, grapes, apples	[88]
			Myricetin	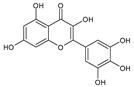	Grapes, berry, apple, strawberry, blueberry, cranberry	[82,89]
		Flavanones	Naringenin	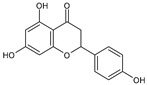	Orange	[84,90]
			Hesperetin	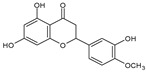	Orange	[84,90]
		Flavan-3-ols	Catechin	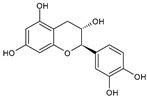	Apples, strawberry, red grapes, sweet cherry, blackcurrant	[80,83,87,91]
			Epicatechin	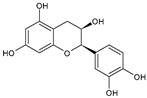	Red grapes, apples, cranberry, cherry	[79,80]
		Flavones	Luteolin	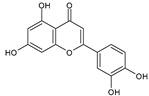	Orange, blackberry	[20,85]
		Anthocyanins	Pelargonidin	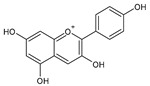	Strawberry, raspberry	[82]
			Delphinidin	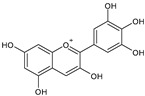	Blueberry, blackcurrant	[82]
			Cyanidin	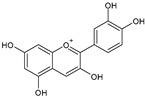	Raspberry, blueberry, cranberry	[82]
			Malvidin	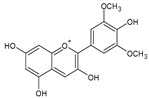	Red grapes, blueberry, cranberry	[82]
			Peonidin	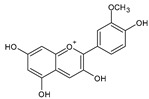	Blueberry, blackcurrant, cranberry	[82,92]
			Petunidin	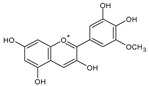	Blueberry, apple	[82,93]
	Stilbenes		Resveratrol	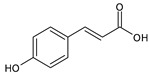	Red grapes, blueberry, cranberry	[31,94]
	Tannins		Ellagic acid	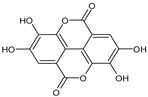	Raspberry, strawberry, blueberry, blackberry	[82,89,95]

**Table 3:** Total phenolics, flavonoids, and anthocyanins content of several fruits.

Fruits	TPC (mg/g Fresh Weight)	TFC (mg/g Fresh Weight)	TAC (mg/g Fresh Weight)	References
*Citrus sinensis*	81.2	-	-	[102]
*Fragaria x. ananassa*	225	-	60–80	[55]
*Malus pumila*	296.3	48.6	-	[102]
*Prunus avium*	88–239	-	82–297	[83]
*Ribes nigrum*	560	46	44	[55]
*Rubus fructicosus*	248–486	276	82–326	[55]
*Rubus idaeus*	126	60	99	[55]
*Vaccinium corymbosum*	261–585	50	25–495	[22]
	525	-	1562.2	[80]
*Vaccinium macrocarpon*	315	157	67–140	[22]
	120–315	-	-	[55]
*Vitis vinifera*	538.6	214.5	-	[55]
	500	-	-	[103]

TPC—Total phenolic content; TFC—Total flavonoid content; TAC—Total anthocyanin content.

**Table 4:** Pharmacological and biological properties of fruits.

Fruits (Botanical Name/Common Name)	Phytoconstituents	Pharmaceutical Properties	References
*Citrus sinensis*/Orange	Rich in vitamin C, A, E, K Rich in α and β-carotene	Antibacterial/fungal, anti-tumoral, antioxidant, anti-microbial, anti-inflammatory, anti-allergic, neuroprotective, decreases cholesterol, promote cardiovascular diseases	[165,166]
*Fragaria x. ananassa*/Strawberry	Rich in vitamin C, anthocyanins Rich in fibers, folic acid, potassium	Anti-inflammatory, antioxidant, anti-diabetic, neuroprotective, anti-microbial, prevent development of cardiovascular diseases	[17,167,168]
*Malus domestica*/Apple	Rich in vitamin C, B_12_, polyphenols Rich in fibers	Anticancer, anti-inflammatory, antioxidant, anti-radiation, boost digestive functions, anti-diabetic, prevent cardiovascular diseases, reduces cholesterol	[169,170,171]
*Prunus avium*/Cherry	Rich in anthocyanins, quercetin, vitamin C Rich in β-carotene	Anti-inflammatory, antioxidant, anti-diabetic, reduces cholesterol, promote cardiovascular diseases	[79,172]
*Ribes nigrum*/Blackcurrant	Rich in vitamin C, A, anthocyanin Rich in potassium, zinc, magnesium, calcium	Antioxidant, anti-inflammatory, anti-cancer, stimulates digestion, reduces blood cholesterol	[87,173]
*Rubus fruticosus*/Blackberry	High in polyphenols, vitamin C, cyanidin-3-glucoside Rich in fibers, folate, manganese. Contains high tannin content	Antiviral/bacterial, antioxidant, antiseptic, anti-aging, anticancer, fights free radical damage, pain reliever	[22,174]
*Rubus idaeus*/Raspberry	Rich in vitamin B, C, anthocyanins, gallic and ellagic acid, ω-3, fibers Rich in iron, copper, potassium, lutein, and folate	Prevents damage caused by free radical, enhances metabolic rate that burns fat, anticancer, antimicrobial, antioxidant	[22,87]
*Vaccinium corymbosum*/Blueberry	Rich in vitamin B, C, A, E, anthocyanins Rich in iron, manganese, selenium, zinc. Contains lutein, β-carotene, and zeaxanthin	Anti-diabetic, antioxidant, anti-inflammatory, anticancer, antimicrobial, reverses aging signs, decreases cholesterol, prevents Alzheimer’s disease	[21,82]
*Vaccinium macrocarpon*/Cranberry	Rich in vitamin A, C, phenols Rich in iron, manganese, magnesium, calcium, and folate	Antibacterial, diuretic, antiseptic, eradicate fat from the lymphatic system, promote cardiovascular health	[175,176]
*Vitis vinifera*/Red grapes	Rich in vitamin C, K, A, B_6_ Rich in potassium, phosphorus, calcium, iron	Anticancer, antioxidant, anti-aging, anti-inflammatory, anti-microbial, hepatoprotective, anti-viral/bacterial, cardioprotective, neuroprotective, reduces blood pressure	[177,178]

**Table 5:** Experimental studies (in-vitro, in-vivo) and human trials showing the pharmacological health benefits of different fruits and their derived products.

Fruit Extracts/Bioactive Compound	Dose Per Day	Experimental Models/Subjects	Duration	Results	References
**Orange**					
In-Vitro					
Red Orange	15, 30 μg/mL	Human keratinocytes (HaCaT) cell line	7 h	▪ Efficiently counteracted UVB-induced response. ▪ Prevent inflammation by inhibiting NF-κB and AP-1 translocation and procaspase-3 cleavage.	[181]
Orange peel extract	400 mg/mL	Human esophageal cancer cell (YM1)	24 h	▪ Reduces the systemic toxicity of chemotherapeutic agents like doxorubicin.	[182]
In-Vivo					
Orange peel extract	0.25%, 0.5%	10 *Apc^Min/+^* mice (4 weeks old) per group	9 weeks	▪ Inhibits intestinal tumor growth.	[183]
Orange extract	100, 200, and 400 mg/mL	N2 wild-type *Caenorhabditis elegans*	5 days	▪ Increased the mean lifespan (dose-dependently). ▪ Improves motility. ▪ Enhanced stress resistance. ▪ Decreased intracellular ROS accumulation.	[184]
Orange peel	50 mg/kg	Xenografts 5 male mice (6–8 weeks old)	14 days	▪ Reduces the size of tumors.	[182]
**Human trials**					
Red orange extract	100 mg	20 Caucasian subjects with skin erythema induced by UV irradiation (aged 26–47 years)	15 days	▪ Significant reduction in the skin erythema degree.	[185]
Red orange extract	100 mg	25 volunteers with tanning skin homogeneity (aged 45–70 years)	5 weeks	▪ Improvement in pigmentation and skin appearance.	[185]
Red orange	50 mg	32 patients with Type 2 diabetes and 28 healthy volunteers	2 months	▪ Decreases serum free radical levels, in patients with high blood oxidative stress status. ▪ Improved blood levels of thiol groups on proteins.	[186]
**Strawberry**					
In-Vitro					
Freeze dried strawberry	NS	Breast (MCF-7 and T47-D) and cervical (CaSki and SiHa) cancer cell lines	48 h	▪ Significantly suppresses the cervical cancer cells but has less effect on breast cancer cells.	[187]
Strawberry rich extracts	25–200 µg/mL	Human oral (KB, CAL-27), prostate (LNCaP), colon (HT-29, HCT-116) and breast (MCF-7) tumor cell lines	24 to 72 h	▪ Inhibits cancer cell proliferation.	[188]
Crude extracts and purified compounds	250 μg/mL (crude extract) and 100 μg/mL (pure compounds)	Human oral (CAL-27, KB), prostate (LNCaP, DU145) and colon (HT-29 and HCT-116) cancer cells	48 h	▪ Inhibits cell proliferation.	[189]
Kaempferol	20, 40, 60 μmol/l	HT-29 colon cancer cells	24 to 72 h	▪ Induces cell cycle arrest. ▪ Inhibit cancer cells growth and proliferation.	[190]
In-Vivo					
Freeze-dried strawberry	2.5%, 5.0% or 10.0%	AOM/DSS induced male Crj: CD-1 mice	20 weeks	▪ Decreases pro-inflammatory mediators and oncogenic signaling pathways.	[191]
**Apples**					
In-Vitro					
Kaempferol	20, 40, 60 μmol/l	HT-29 colon cancer cells	24 to 72 h	▪ Induces cell cycle arrest. ▪ Inhibit cancer cells growth and proliferation.	[190]
Quercetin	5, and 10 μg/mL	HT1080 human fibrosarcoma cells	24 h	▪ Suppresses phenazine methosulfate (PMS)-induced intracellular ROS formation. ▪ Decreases matrix metalloproteinase activity. ▪ Reduced cell motility.	[192]
In-Vivo					
Whole apple extract	2.5, 5 and 10 mg/mL	*Caenorhabditis elegans*	4 days	▪ Slowdowns the aging process. ▪ Enhances stress-resistance. ▪ Extends lifespan	[193]
Apple peel extract	NS	8 male C57BL/6J mice (6 weeks old)	10 weeks	▪ Decreases blood glucose concentration.	[194]
**Cherry**					
In-Vitro					
Cherry extracts	0.025, 0.05, 0.25, and 0.5% dry wt.	Colon (HT-29) and breast (MCF-7) cancer cells	24 h	▪ Exerts anti-proliferative effect on cancer cells.	[195]
In-Vivo					
Cherry extract		8 male C57BL/6J mice (6 weeks old)	10 weeks	▪ Decreases blood glucose concentration.	[194]
**Blackcurrant**					
In-Vitro					
Blackcurrant extracts	0.025, 0.05, 0.25, and 0.5% dry wt.	Colon (HT-29) and breast (MCF-7) cancer cells	24 h	▪ Exerts anti-proliferative effect on cancer cells.	[195]
Blackcurrant press residue extracts	20, 75, 100, or 125 μg GAE/mL	Caco-2, HT-29, and HCT-116 cells	24 to 48 h	▪ Suppress proliferation of cancer cell.	[196]
**Human trials**					
FL and CAM30 prepared from blackcurrant extract	Both products contain 672 mg blackcurrant powder	30 healthy volunteers (16 women, 14 men, Aged 20–60 years)	2 weeks	▪ Expresses anticancer activity by decreasing the activity of the bacterial β-glucuronidase enzyme and lowering the fecal pH.	[197]
**Blackberry**					
In-Vitro					
Blackberry extract	25–200 µg/mL	Human oral (KB, CAL-27), prostate (LNCaP), colon (HT-29, HCT-116) and breast (MCF-7) tumor cell lines	24 to 72 h	▪ Exerts anti-proliferative effects.	[188]
Anthocyanin-rich extracts from hull and crude blackberry	13.6 to 49.2 µg of monomeric anthocyanins/mL	HT-29 colon tumor cells	48 to 72 h	▪ Induces significant antioxidant and anti-proliferative activity.	[198]
In-Vivo					
Freeze-dried blackberry fruits	50 g/kg	Fisher 344 male rats (3–4 years old)	13 weeks	▪ Inhibits colonic aberrant crypt foci (ACF) formation.	[199]
**Raspberry**					
In-Vitro					
Red raspberry extracts	5%, 7.5%, and 10%	AGS stomach, LoVo colon cancer cells, and MCF-7 breast cancer cell lines	24 to 48 h	▪ Reduces the survival of cancer cells.	[200]
Red raspberry	10, 25, 50 mg/mL	Human hepatocellular carcinoma HepG2 and Huh7 cell lines	24 to 96 h	▪ Inhibits growth and proliferation of hepatocellular carcinoma (HCC) cells.	[201]
In-Vivo					
Fresh-dried raspberry	29.0 g	Wild-type male C57BL/6J mice (6 weeks old)	10 weeks	▪ Reduces disease activity index (DAI) score and risk of colorectal cancer (CRC) development.	[202]
Freeze-dried raspberry extracts	100, 200, 300 mg/kg	C57BL/6J male mice (6 weeks old)	8 weeks	▪ Reduces hyperlipidemia and oxidative stress. ▪ Reduces Hmgcr expression which results in low cholesterol	[203]
**Blueberry**					
In-Vitro					
Freeze-dried strawberry	NS	Breast (MCF-7 and T47-D) and cervical (CaSki and SiHa) cancer cell lines with different requirements for estrogen	48 h	▪ Significantly suppresses the cervical cancer cells and T47-D breast cancer cells.	[187]
Dried extracts and fractions	50–10,000 µg/mL	HT-29 and Caco-2 colon cancer cell lines	48 h	▪ Inhibits cancer cell proliferation. ▪ Induces apoptosis.	[204]
Blueberry extracts	25–200 µg/mL	Human oral (KB, CAL-27), prostate (LNCaP), colon (HT-29, HCT-116) and breast (MCF-7) tumor cell lines	24 to 72 h	▪ Inhibits cancer cell proliferation.	[188]
Pterostilbene	50 µM	Human colon carcinoma HT-29 cells	4 h	▪ Suppresses inflammation. ▪ Suppresses cell growth.	[205]
Blueberry	NS	Fischer 344 male rats	10 weeks	▪ Improved HSP70-mediated protection against a number of neurodegenerative processes in the brain.	[206]
In-Vivo					
Blueberry extracts	5 mg/mL	*Drosophila melanogaster*	76 days	▪ Increased the mean lifespan of fruit flies by 10%. ▪ Up-regulates the gene expression of catalase (CAT), superoxide dismutase (SOD), and Rpn11 and down-regulates Methuselah (MTH) gene.	[207]
Blueberry extracts	50, 100, and 200 mg/mL	*Caenorhabditis elegans*	7 days	▪ Increased the mean lifespan. ▪ Enhanced stress resistance. ▪ Improved health indexes.	[180]
Freeze-dried blueberry extracts	50 g/kg	Fisher 344 male rats (3–4 years old)	13 weeks	▪ Reduces azoxymethane (AOM)-induced aberrant crypt foci (ACF) formation. ▪ Increases hepatic glutathione-S-transferase (GST) activity.	[199]
Pterostilbene	40 p.p.m. (0.004%)	AOM induced Fisher 344 male rats	45 weeks	▪ Reduces tumor multiplicity, by inhibiting the Wnt/β-catenin signaling pathway.	[205]
Blueberry juice	NS	12 C57BL/6 male mice (4 weeks old)	12 weeks	▪ Improved insulin resistance. ▪ Reduced body weight. ▪ Reduced serum glucose levels. ▪ Exerted potentially anti-inflammatory effect.	[208]
**Cranberry**					
In-Vitro					
Crude cranberry extract	1, 10, 50 and 100 µg/mL	HT-29 human colon adenocarcinoma cells	6 h	▪ Decreased the risk of colon cancer by suppressing inflammatory responses.	[209]
Quercetin	1, 10, 50, and 100 µmol/L	HT-29 human colon adenocarcinoma cells	6 h	▪ Reduces the risk of colon cancer.	[209]
In-Vivo					
Flavonoid-rich fraction 6 (Fr6) and purified proanthocyanidin (PAC)	100 mg/kg proanthocyanidin (PAC) and 250 mg/kg Fr6	Xenografts Balb/c female mice (4–6 years old)	Every 2 days for 3 weeks	▪ Reduces tumor growth and volume.	[210]
Cranberry extracts and dried cranberry	0.1% cranberry extract and 1.5% dry cranberry	Dextran sulfate sodium (DSS) induced murine colitis	1 week	▪ Prevent colitis. ▪ Decreases inflammatory cytokines.	[211]
**Human trials**					
Cranberry juice	125, 250 and 500 mL/day	30 abdominally obese men	4 weeks	▪ Increased plasma HDL-cholesterol concentrations.	[212]
Cranberry juice cocktail	202 mL/day	Adults (≥19 years)	2 days	▪ Lowered C-reactive protein level. ▪ Improved cardio metabolic profiles.	[213]
Cranberry juice	480 mL/day	30 women, 26 men	8 weeks	▪ Improved CVD risk factor including C-reactive protein, diastolic blood pressure, insulin resistance, circulating triglycerides and glucose.	[214]
Cranberry beverage	450 mL/day	78 overweight or obese men and women	8 weeks	▪ Improved antioxidant status. ▪ Reduces CVD risk factors. ▪ Increased HDL-cholesterol levels.	[215]
**Red grapes**					
**Human trials**					
Red grape cell powder	200 mg, 400 mg/day	Fifty adults (35 male, 15 female) with pre- and mild-hypertension (≥35 and <70 years)	12 weeks	▪ Decreased diastolic blood pressure and oxidative stress.	[216]
Red grape juice	100 mL/day	15 healthy and 26 hemodialysis patients	14 days	▪ Improved the lipoprotein profile. ▪ Decreases total cholesterol (TC) and oxidized-LDL concentrations.	[217]
Red grape juice	100 mL/day	32 hemodialysis patients	14 days	▪ Increased HDL-cholesterol concentrations. ▪ Reduced plasma concentrations of total cholesterol and apolipoprotein B (apoB).	[98]
Red grape juice	300 mL/day	26 healthy and non-smokers males	1 month	▪ Increased serum HDL-cholesterol and apolipoprotein B (apoB) levels. ▪ Decreased homocysteine (Hcy) levels.	[218]

NS—not specified.
